# Distinct profiles of mitochondrial bioenergetics and redox balance in left atrial and ventricular myocardium in the healthy rat heart

**DOI:** 10.1113/EP093102

**Published:** 2025-11-04

**Authors:** Tingting Fang, Alex Chan, Janice Chew‐Harris, Toan Pham

**Affiliations:** ^1^ Auckland Bioengineering Institute The University of Auckland Auckland New Zealand; ^2^ Christchurch Heart Institute The University of Otago Christchurch New Zealand

**Keywords:** ATP, catalase, left atrium, left ventricle, mitochondrial function, ROS, SOD

## Abstract

The left ventricle (LV) is the primary pumping chamber of the heart, generating high systolic pressure to sustain systemic circulation. LV contractile dysfunction is a hallmark of various cardiovascular diseases and is associated with mitochondrial dysfunction, characterised by decreased oxidative phosphorylation (OXPHOS) capacity and increased oxidative stress. While our understanding of cardiac mitochondrial physiology has been gained from studies on LV tissues in animal models or atrial tissues in human studies, findings are often generalised across cardiac regions. Given that fundamental differences in anatomical structure, physiological function and metabolic demands exist between the LV and left atrium (LA), this study aimed to compare mitochondrial bioenergetics between LV and LA tissues from healthy rat hearts. Using high‐resolution respirometry coupled with fluorimetry, we assessed mitochondrial respiration, ATP production and hydrolysis, and reactive oxygen species (ROS) production rates. Protein expression of mitochondrial respiratory complexes and antioxidant enzymes was quantified using western blotting. Our results showed that per tissue mass, LV tissues exhibited greater mitochondrial OXPHOS respiration, ATP production and hydrolysis rates, ROS production rate, and higher protein levels of mitochondrial complexes and antioxidant enzymes, consistent with higher citrate synthase activity as a marker of mitochondrial content. However, when normalised to mitochondrial content, LV tissues exhibited lower OXPHOS respiration and ATP production, expression of mitochondrial complexes and antioxidant proteins compared to LA. This study provides new insights into chamber‐specific differences in mitochondrial function under physiological conditions, suggesting the importance of considering regional mitochondrial profiles in studies of cardiac mitochondrial function in health and disease.

## Abstract (highlights)

- **What is the central question of this study?**
  How do mitochondrial energetics and redox balance differ between the left ventricular and left atrial myocardium in healthy rat hearts?
- **What is the main finding and its importance?**
  Left ventricular tissues demonstrate higher mitochondrial respiratory capacity per tissue mass, but lower function when normalised to mitochondrial content compared to left atrial tissues. The heart chamber‐specific differences highlight the need to study region‐specific mitochondrial profiles in both health and disease.

## 1 INTRODUCTION

The heart relies on a continuous high‐energy supply from mitochondria to maintain high workloads of contractile function (Kim & Choi, 2021). With high energy demands, the heart has the highest density of mitochondria across the body, where mitochondria occupy 35–40% of cardiomyocyte volume (Mohamud et al., 2023). Mitochondria produce 95% of ATP through oxidative phosphorylation (OXPHOS) (Pham et al., 2014; Sun et al., 2024) to meet the heart's substantial daily consumption of 3.5–5 kg of ATP (Neubauer, 2007).

Reactive oxygen species (ROS) are formed as byproducts of mitochondrial respiration pathways (Sarniak et al., 2016), generated primarily by electron leakage from the mitochondrial electron transport system (Aon et al., 2010). Cardiomyocytes possess a robust endogenous antioxidant system, which includes several key antioxidant enzymes such as catalase and superoxide dismutase. These two enzymes synergistically convert superoxide radicals and hydrogen peroxide into harmless water and oxygen, to prevent excessive oxidative stress damage and maintain ROS at controlled levels (Galasso et al., 2021; Lee et al., 2018; Miao & St Clair, 2009). Under normal conditions, levels of ROS within physiological ranges regulate several critical cellular processes and act as intracellular signalling, including stress response (Merry et al., 2020), cardiac development and maturation, calcium handling, and excitation–contraction coupling (Gong et al., 2025; Kubin et al., 2011; Momtahan et al., 2019). However, chronic excess ROS production and impaired redox balance are implicated in the pathogenesis of multiple cardiovascular diseases, including myocardial hypertrophy, hyperlipidaemia, ischaemia–reperfusion injury, arrhythmias and diabetic cardiomyopathy (Hulsmans et al., 2012; Li et al., 2025; Nishikawa et al., 2007).

The left atrium (LA) and left ventricle (LV) exhibit differences in structural and functional characteristics in the mammalian heart. The LA acts primarily as a low‐pressure reservoir for pulmonary venous return and develops low pressure for ventricular filling (Ahlberg et al., 2021). In contrast, the LV develops higher systolic pressure to pump oxygenated blood into systemic circulation. This higher workload is consistent with a thicker free wall of the LV, higher mitochondrial density, higher oxidative metabolism capacity, and extensive coronary perfusion to maintain a high oxygen supply to contracting cardiomyocytes (Cryer & Bartley, 1974; Scheiber et al., 2019).

Comparative studies assessing mitochondrial function between heart‐specific regions remain scarce. Earlier findings on patients with ischaemic or idiopathic dilated cardiomyopathy reported reduced OXPHOS capacity in the LV endocardium compared to the epicardium (Sharov et al., 2000). Mitochondrial respiration in the LV was shown to be 50% higher than in the right atrium (RA) when supported by octanoyl‐carnitine substrate (Scheiber et al., 2019). Mitochondria from the right ventricle (RV) of rat hearts produced more ROS than those from the LV (Schlüter et al., 2018; Schreckenberg et al., 2015), although catalase protein expression and activity appeared similar across chambers in the rat heart and non‐failing human heart (Liu et al., 2008; Manni et al., 2016). These findings suggest potential functional heterogeneities in mitochondrial respiration and ROS regulation between cardiac chambers.

Interestingly, the differences in mitochondrial function between the LA and LV remain poorly characterised. At the tissue level, it has been reported that the LV muscle exhibits a lower isometric tension cost compared to right atrial muscles, implying that the LV muscle may require lower ATP consumption to generate the same isometric contraction force with greater energetic economy (Narolska et al., 2005). To our knowledge, no study has compared mitochondrial energy efficiency at the mitochondrial level between the LV and LA tissues. This study provided the first characteristics of mitochondrial respiratory function, ATP production and ATP hydrolysis rate, ROS production rate, protein levels of respiratory electron complexes and endogenous antioxidant enzymes in the LA versus LV of healthy rat hearts. We also assessed mitochondrial adaptation under simulations of ischaemia–reperfusion conditions in vitro. Our findings provide a foundational understanding of region‐specific mitochondrial function in the heart and offer potential implications for future studies on heart disease and therapeutic interventions.

## 2 METHODS

### 2.1 Ethical approval

All animal handling procedures were conducted in accordance with approved protocols by the University of Auckland Animal Ethics Committee (AEC22653). Male Wistar rats (250–350 g, *n* = 11) were purchased from the animal facility (Vernon Jansen Unit) at the University of Auckland. The rats were housed in pairs in a 12/12 h light–dark cycle at 21°C. Standard rat chow and tap water were available ad libitum. Each rat was brought to our department (Auckland Bioengineering Institute within the University of Auckland) and placed in a climate‐controlled chamber with access to food and water for at least 1 h to minimise any stress arising from the transportation.

### 2.2 Tissue preparation

On each experimental day, a rat was deeply anaesthetised by isoflurane inhalation (5% in O_2_) and administered an injection of heparin (1000 IU/kg). Following a cervical dislocation, the excised heart was rapidly plunged into a cold Tyrode solution and Langendorff‐perfused with oxygenated Tyrode solution at room temperature to remove leftover blood in the coronary circulation of the heart. The solution contained (in mmol/L) 130 NaCl, 6 KCl, 1 MgCl_2_, 0.3 CaCl_2_, 0.5 NaH_2_PO_4_, 10 Hepes, 10 glucose, and 20 2,3‐butanedione monoxime, with pH adjusted to 7.4 using Tris. LV and LA tissues were dissected from the same heart to ensure paired comparisons.

Mitochondrial respiration was measured in freshly prepared tissue samples, and the remaining tissues were stored at −80°C for later protein analysis. Fresh tissues (∼20 mg) were quickly blotted on filter paper, weighed and transferred to 500 µL of cold MiRO5 assay buffer (in mmol/L: 110 sucrose, 60 potassium lactobionate, 20 Hepes, 20 taurine, 10 KH_2_PO_4_, 0.5 EGTA, and 1 g/L fraction V BSA free fatty acid, pH 7.1 at 30°C). Heart samples were then carefully minced with scissors, followed by further homogenisation for 10 s at medium speed using a tissue homogeniser (Ommi International, Hennesaw, GA, USA). Homogenates were kept on ice for subsequent mitochondrial experiments, and the remaining samples were stored at −80°C for later enzymatic analysis.

### 2.3 Mitochondrial respiration

A high‐resolution respirometer (O2k, Oroboros Instruments, Innsbruck, Austria) was used to measure mitochondrial function. The O2k comprises two independent 2‐mL chambers, each equipped with polarographic oxygen sensors and stoppers that facilitate substrate, uncoupler and inhibitor titrations. A detachable fluorimeter (Oroboros Instruments) was inserted into the front window of each O2k chamber to measure the fluorescence of different fluorophores simultaneously alongside the O_2_ consumption rate or flux. The O_2_ concentration of the assay medium was 195 µmol/L at 95 kPa barometric pressure. All experiments were performed at 37°C.

#### 2.3.1 Protocol A: measurements of ATP production and hydrolysis rates

Alongside measurement of oxygen flux, ATP production and hydrolysis rates were measured using Magnesium Green (MgG, pentapotassium salt, cell impermeant, M3733, Thermo Fisher Scientific, Waltham, MA, USA) fluorescence at excitation and emission wavelengths of 470 and 520 nm, respectively (Power et al., 2014, 2016). ATP protocol was performed using the standard buffer MiRO5, without MgCl_2_. MgG (5 µmol/L) was added to the medium, and blebbistatin (50 µmol/L) was used as an inhibitor of the myosin heavy chain. MgCl_2_ (1 mmol/L) was added to each chamber to calibrate the MgG signal. Exactly 1.5 mg of LV or 3 mg of LA homogenates was added to the chambers and equilibrated. NADH‐pathway‐linked leak respiration was determined using malate (2 mmol/L), glutamate (10 mmol/L), and pyruvate (5 mmol/L). Succinate (10 mmol/L) was added to assess NADH‐ and FADH_2_‐pathway‐linked leak respiration; despite the presence of a small amount of ADP (∼0.03 µmol/L) in homogenate samples, it might contribute to negligible OXPHOS respiration. Excess ADP (2.5 mmol/L) was added to stimulate NADH‐ and FADH_2_‐pathway linked OXPHOS respiration. The tissue was allowed to respire in anoxia (i.e. O_2_ concentration level was zero) and remained in this state for 40 min to mimic the oxygen depletion in ischaemic conditions in vivo. O_2_ concentration was then restored to normal by opening stoppers to allow for air equilibration. Oligomycin (2 µmol/L), an ATP synthase inhibitor, was added to stop mitochondrial ATP production and return the MgG signal flux to baseline within 15 min. Antimycin A (1 µmol/L) was added to inhibit complex III to determine any residual O_2_ flux.

#### 2.3.2 Protocol B: measurements of H_2_O_2_ production rates

The net H_2_O_2_ production rate was simultaneously measured using Amplex UltraRed (AUR, A36006, Thermo Fisher Scientific) in the presence of horseradish peroxidase (HRP) and superoxide dismutase (SOD). The superoxide radicals released from mitochondria are reduced to form H_2_O_2_ by the addition of exogenous SOD. The combined mitochondrial H_2_O_2_ and exogenous SOD‐derived H_2_O_2_ are linked to HRP, which in turn reacts with AUR to form a resorufin as a fluorescent product with excitation and emission wavelengths of 525 and 550 nm, respectively (Broome et al., 2022; Hedges et al., 2020). AUR (25 µmol/L), HRP (10 U), and SOD (10 U) were added to each chamber. H_2_O_2_ signal was calibrated with titration of H_2_O_2_ (0.122 µmol/L) three times and allowed to equilibrate before adding samples to the chamber. MgCl_2_ (1 mmol/L) and homogenate samples were added to the chamber, followed by an equilibration period. Malate (2 mmol/L), glutamate (10 mmol/L), pyruvate (5 mmol/L) and succinate (10 mmol/L) were supplied to initiate NADH‐ and FADH_2_‐linked leak respiration. Saturated ADP (2.5 mmol/L) was added to stimulate the OXPHOS respiratory state. Tissues were respired in anoxic conditions for 40 min and then reoxygenated to mimic reperfusion conditions. Multiple titrations of the uncoupler carbonyl cyanide *m*‐chlorophenyl hydrazone (CCCP, 0.5 µmol/L) induced uncoupled respiration as a measure of maximal electron transport system capacity. Antimycin A (1 µmol/L) was added to inhibit complex III to determine the residual O_2_ flux.

### 2.4 Citrate synthase activity

Stored frozen homogenate samples were thawed, vortexed and centrifuged at 14,000 *g* for 10 min at 4°C. Collected supernatants were used to determine citrate synthase (CS) activities. The fluorescence absorbance was measured at 412 nm on a BioTek microplate reader (BioTek Instruments, Winooski, VT, USA) at 25°C in the presence of 0.5 mmol/L oxaloacetate in Tris–HCl (50 mmol/L) buffer pH 8 that contained acetyl CoA (0.1 mmol/L) and 5,5‐dithiobis‐(2‐nitrobenzoic acid) (0.2 mmol/L). The slope of CS absorbance change was calculated using an extinction coefficient of 13.6 L/(mmol cm) and normalised to the corresponding protein content. Total soluble protein content from the same supernatants was measured using a Pierce BCA protein assay kit (Thermo Fisher Scientific).

### 2.5 Immunoblotting analysis

Frozen intact tissue samples (∼40 mg) were mechanically minced in a 15‐fold volume of cold RIPA lysis buffer (in mmol/L, 50 Hepes pH 7.4, 150 NaCl, 10% glycerol, 1.5 MgCl_2_, 1 EGTA, 1 sodium orthovanadate, 1% Triton X‐100, 1% sodium deoxycholate, 0.1% SDS) supplemented with protease inhibitor cocktail (complete mini EDTA‐free protease inhibitor, Roche, Mannheim, Germany), and 2 mmol/L phenylmethylsulfonyl fluoride. Samples were homogenised using a Qiagen (Qiagen, Dusseldorf, Germany) Tissue Lyser II at 30 Hz for 2 min and then rested on ice for 1 h before being centrifuged at 21,000 *g* at 4°C for 10 min. The supernatant was collected and diluted to determine protein concentrations using a Pierce BCA protein assay kit. Samples were prepared for western blotting by diluting to 3 g/L with 4× Laemmli buffer (10% glycerol, 2% SDS, 0.25% bromophenol blue, 400 mmol/L dithiothreitol (DTT), 0.5 mol/L Tris–HCl (pH 6.8)). An aliquot was removed from the sample preparation and stored under non‐denaturing conditions to preserve the complex IV band when probing for OXPHOS, while the remaining sample volume was boiled at 95°C for 5 min, as per the standard procedure. The supernatant proteins were determined by SDS‐PAGE and processed for immunoblotting using standard procedures described previously (Pham et al., 2020). Protein bands on polyvinylidene fluoride membranes were visualised using enhanced chemiluminescent detection reagent in a ChemiDoc system (Bio‐Rad Laboratories, Hercules, CA, USA). Band densitometries were measured using ImageJ software (National Institutes of Health, Bethesda, MD, USA), and band intensities were normalised to total protein expression on the same membrane, as determined by amido black staining, and a reference sample for comparison between gels. Antibodies were purchased from Abcam (Waltham, MA, USA): total OXPHOS antibody cocktail (1:2000, ab110413), catalase (1:500, ab16731) and SOD2 (1:5000, ab13534).

### 2.6 Data analyses

All respirometric and fluorometric data were recorded and analysed offline using *DatLab* 7.1 software (Oroboros Instruments). All respiration rates were corrected for residual O_2_ flux. ATP flux data were calibrated using separate assays without tissue samples, previously described (Pham et al., 2014). ATP flux data were corrected with ATP synthase inhibition by adding oligomycin. The P/O ratio was determined by ATP flux relative to O_2_ flux during OXPHOS respiratory state under normoxic conditions. To account for non‐mitochondrial H_2_O_2_ production, H_2_O_2_ flux data were corrected by subtracting the background H_2_O_2_ signal measured before homogenate sample additions. All data were normalised to tissue wet mass and CS activity.

### 2.7 Statistical analyses

Statistical analyses were performed using GraphPad Prism version 10.0 software (GraphPad Software, Boston, MA, USA). Two‐way analysis of variance and Fisher's least significant difference *post hoc* test were used to analyse only OXPHOS protein values between groups. Student's *t*‐test was used to test the differences between the two groups. All data are presented as means ± SD. A significant difference was considered when *P* < 0.05. Prism software was used to generate graphs.

## 3 RESULTS

### 3.1 Mitochondrial O_2_ fluxes are comparable in the LA and LV homogenates

**FIGURE 1:**
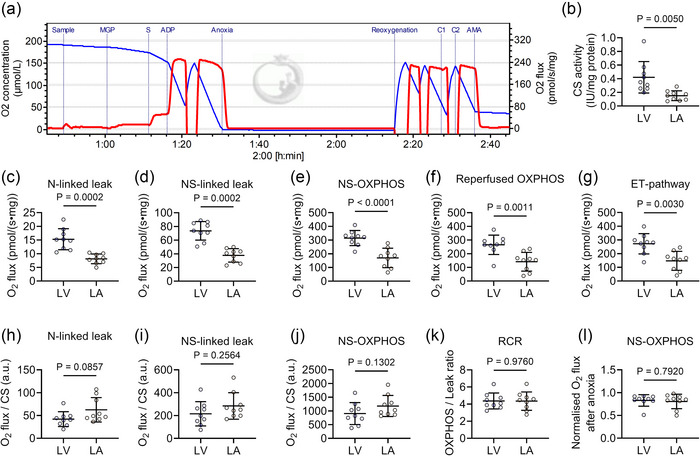
Mitochondrial O_2_ fluxes are comparable in the LA and LV homogenates. (a) Representative raw traces of O_2_ concentration (blue) and O_2_ flux (red) from LA mitochondria throughout the experimental Protocol B and respiratory states of each combination of substrates (malate, glutamate, pyruvate, succinate), uncoupler (CCCP) and inhibitors (antimycin A). G, glutamate; P, pyruvate; M, malate; S, succinate; AMA, antimycin A; CCCP, carbonyl cyanide *m*‐chlorophenyl hydrazone; N‐linked, NADH‐linked; NS‐linked, NADH‐ and FADH_2_‐linked; OXPHOS, oxidative phosphorylation. (b) Levels of CS activity. (c–g) O_2_ flux per tissue mass at various respiratory states. (h–j) O_2_ flux per CS activity. (k) Respiratory control ratio. (l) Normalised O_2_ flux at OXPHOS state before and after 40 min anoxia. LV (*n* = 9), LA (*n* = 9). Data are expressed as means ± SD.

Two substrate–uncoupler–inhibitor titration protocols were employed to evaluate mitochondrial respiratory capacity and coupling control. Figure 1a illustrates a representative raw trace of O_2_ flux from Protocol B. CS activity, an indicator of mitochondrial content, was significantly higher in LV tissues compared to LA tissue (*P *= 0.0050, Figure 1b). Leak respiration rate reflects O_2_ consumption to counteract ion leaks in the absence of ATP production, facilitated by electron transport through NADH‐ and FADH_2_ pathways. OXPHOS respiration quantifies O_2_ flux coupled to ATP synthesis. CCCP dissipates the proton gradient, preventing proton accumulation in the intermembrane space, thereby driving maximal electron transfer capacity through uncoupling respiration. When normalised to tissue mass, mitochondrial O_2_ flux across all respirometry states was consistently higher in the LV tissue compared to the LA tissue (*P *= 0.0002, *P* = 0.0002, *P *< 0.0001, *P *= 0.0011, *P *= 0.0030, respectively, Figure 1c–g).

When normalised to CS activity as a standard method to account for mitochondrial density in intrinsic mitochondrial function data, mitochondrial O_2_ flux respiration in NADH‐linked leak (*P* = 0.0857) and NAHD–FADH_2_‐linked OXPHOS (*P* = 0.1302) respiratory states was not significantly different between groups (Figure 1h–j). Respiratory control ratio (RCR) was calculated as the ratio of O_2_ flux during the OXPHOS state relative to the leak respiratory state to determine mitochondrial coupling efficiency. The RCRs were similar between LV and LA mitochondria (*P* = 0.9760, Figure 1k). An anoxia–reoxygenation protocol was used to challenge mitochondrial recovery capacity and evaluate their resilience or vulnerability under compromised conditions. Following 40 min of anoxia, mitochondrial O_2_ flux in the reperfusion state was significantly decreased by 20% in both LV and LA mitochondria, with no difference in O_2_ flux decline between the two groups (*P* = 0.7920, Figure 1l). These results indicate that mitochondrial respiration per tissue mass is higher in the LV compared to the LA due to higher mitochondrial content, while intrinsic mitochondrial respiration in the LA is higher per mitochondrion unit.

### 3.2 LA mitochondria exhibit higher ATP flux despite a similar P/O ratio

**FIGURE 2:**
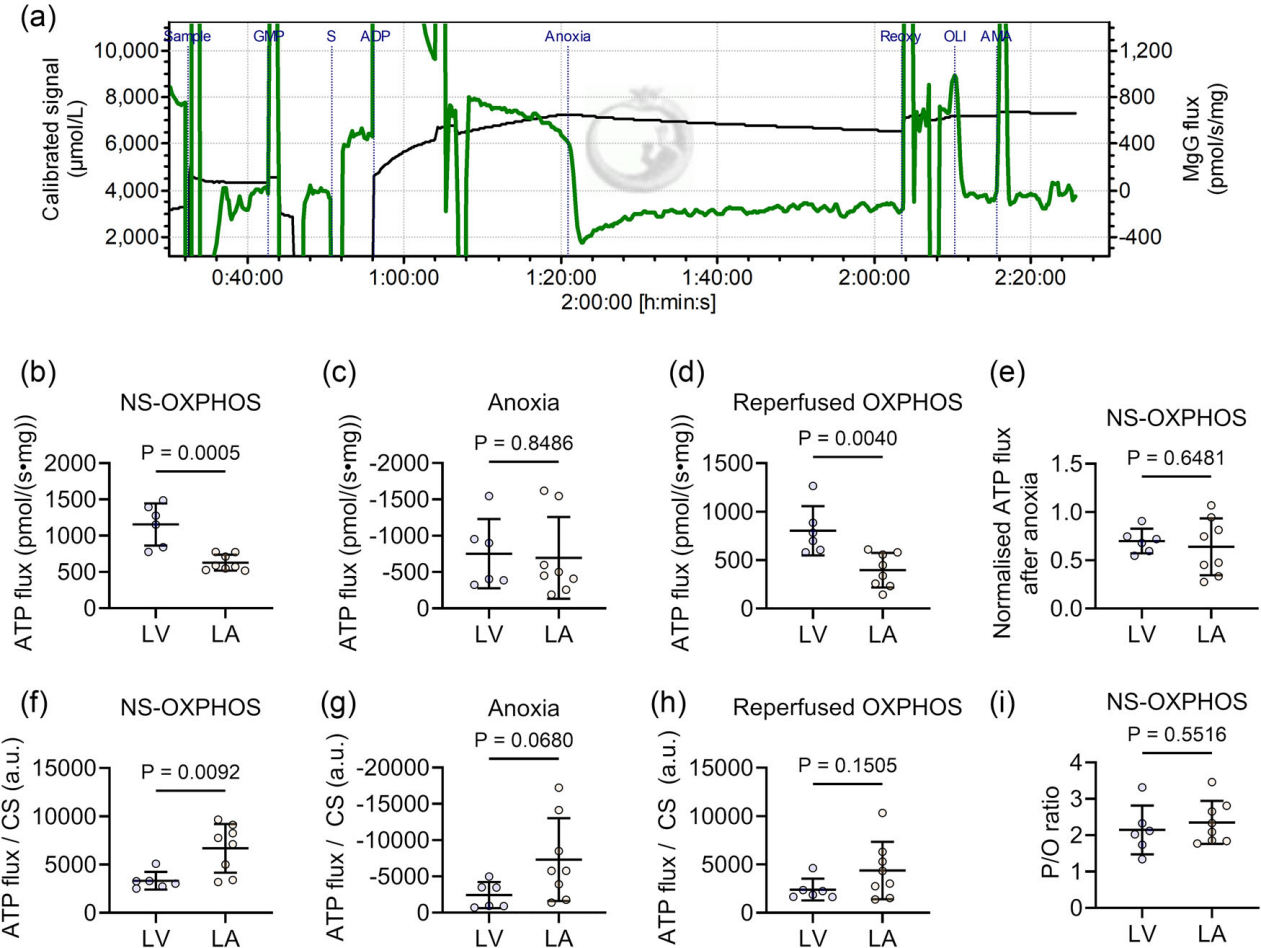
LA mitochondria exhibit higher ATP flux despite a similar P/O ratio. (a) A representative raw trace of MgG signal (black) and MgG or ATP flux (green) in LA mitochondria from Protocol A. G, glutamate; P, pyruvate; M, malate; S, succinate; AMA, antimycin A; NS‐linked, NADH‐ and FADH_2_‐linked; Oli, oligomycin; OXPHOS, oxidative phosphorylation. (b–d) ATP flux per tissue mass at various respiratory states. (e) Normalised ATP flux at OXPHOS state after 40 min of anoxia. (f–h) ATP production rate per CS activity at various respiratory states. (i) P/O ratio. LV (*n* = 6), LA (*n* = 8). Data are expressed as means ± SD.

Figure 2a illustrates a schematic trace of MgG fluorometry measurement in LV mitochondria from Protocol A. When normalised to tissue mass, the steady‐state ATP production rate at the OXPHOS (*P* = 0.0005, Figure 2b) and reperfused OXPHOS states (*P* = 0.0040, Figure 2d) was significantly higher in LV tissue compared to LA tissue. Following 40 min of anoxia, ATP flux was decreased by 40% during the reperfusion state, and there was no difference in ATP flux decline between groups (*P* = 0.6481, Figure 2e). During anoxia, ATP synthase can be reversed to consume ATP, and hence a negative ATP flux was detected in the experimental protocol. The capacity of mitochondrial ATP synthase to hydrolyse ATP during anoxia was similar between groups (*P* = 0.8486, Figure 2c). When normalised to CS activity (Figure 2f–h), LV mitochondria showed a lower steady‐state ATP production rate only at the OXPHOS state compared to LA mitochondria (*P* = 0.0092, *P* = 0.0680, *P* = 0.1505, respectively). The P/O ratio, representing mitochondrial energy efficiency in producing ATP per oxygen molecule consumed, showed no difference between LV and LA mitochondria (*P* = 0.5516, Figure 2i).

### 3.3 H_2_O_2_ emission is equivalent in LA and LV mitochondria

**FIGURE 3:**
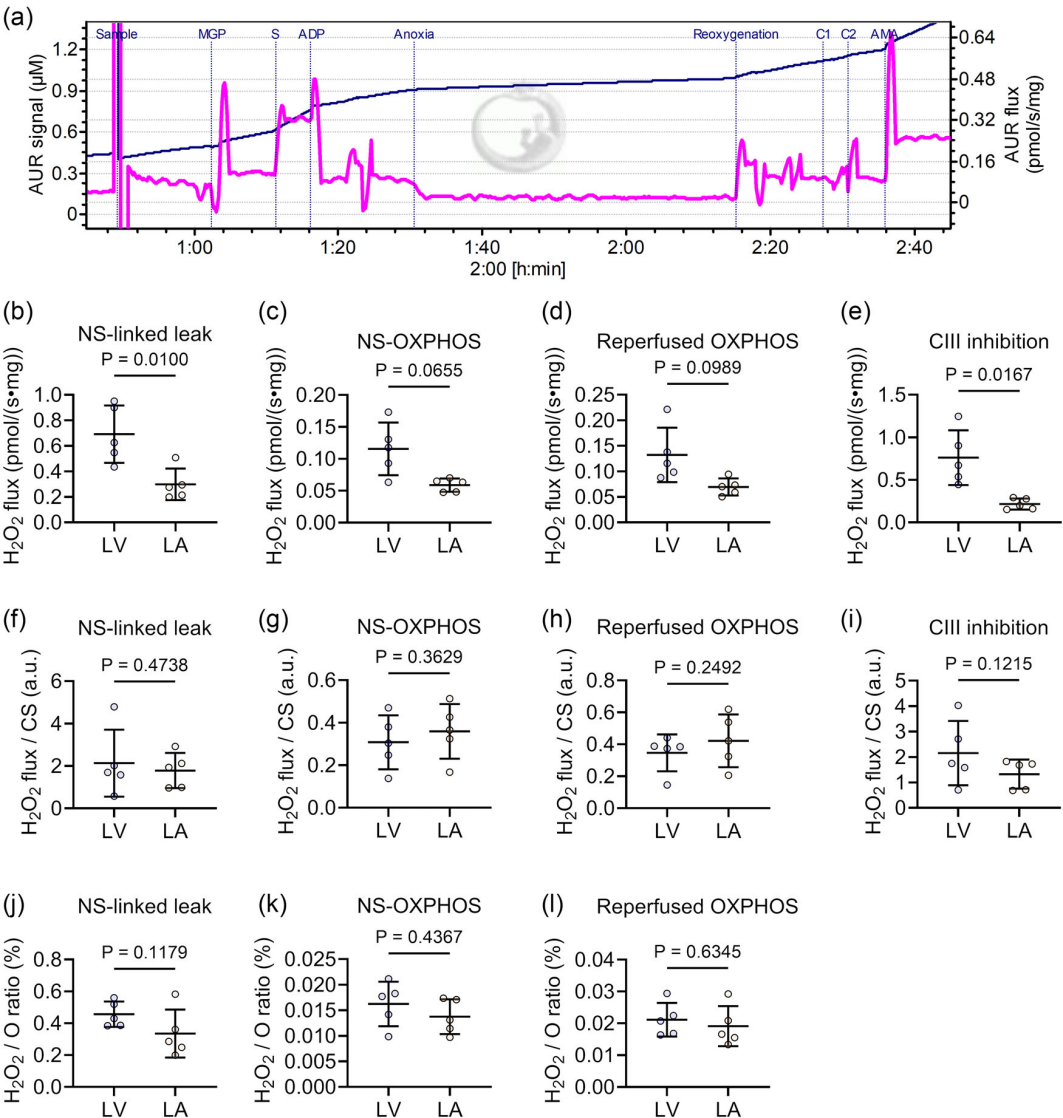
H_2_O_2_ emission is equivalent in LA and LV mitochondria. (a) A representative raw trace of AUR signal (black) and H_2_O_2_ flux (pink) in LA mitochondria from Protocol B. G, glutamate; P, pyruvate; M, malate; S, succinate; C, uncoupler; AMA, antimycin A; NS‐linked, NADH‐ and FADH_2_‐linked; OXPHOS, oxidative phosphorylation. (b–e) H_2_O_2_ flux per tissue mass at various respiratory states. (f–i) H_2_O_2_ flux per CS activity. (j–l) H_2_O_2_ flux relative to O_2_ flux (H_2_O_2_/O%). LV (*n* = 5), LA (*n* = 5). Data are expressed as means ± SD.

H_2_O_2_ flux measurements in Protocol B are presented schematically in Figure 3a. H_2_O_2_ production per tissue mass was substantially higher in the LV group in the non‐phosphorylating leak respiratory state (*P* = 0.0100, Figure 3b), but no difference was found in the OXPHOS respiratory state (*P *= 0.0655, Figure 3c). However, when normalised to CS activity, H_2_O_2_ production did not differ between the two groups (*P *= 0.4738, *P *= 0.3629, *P *= 0.2492, *P *= 0.1215, respectively, Figure 3f–i). Similarly, ROS production expressed as a percentage of O_2_ flux (H_2_O_2_/O%) did not differ between groups (*P *= 0.1179, *P *= 0.4367, *P *= 0.6345, respectively, Figure 3j–l). These results suggest no differences in mitochondrial H_2_O_2_ generation after normalisation for CS activity, indicating that the higher H_2_O_2_ generation in the LV per tissue mass was due to a higher mitochondrial number.

### 3.4 Higher respiratory chain complex and antioxidant enzyme protein levels in LA vs. LV mitochondria

**FIGURE 4:**
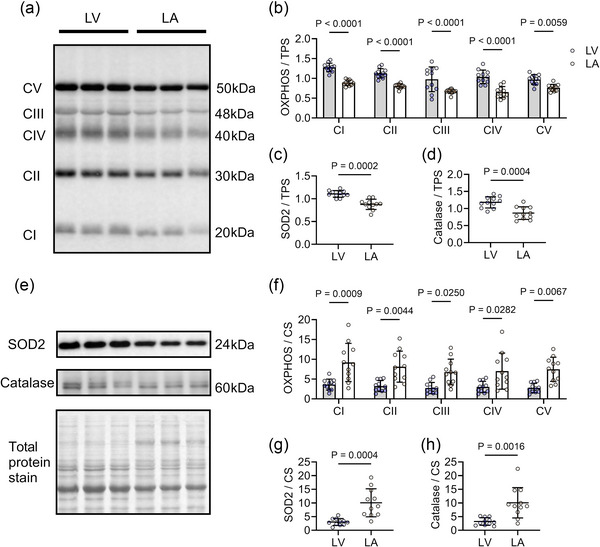
Higher respiratory chain complex and antioxidant enzyme protein levels in LA versus LV mitochondria. (a, b, f) Protein expression of OXPHOS complexes I–V (CI–CV) normalised to total protein stain (a, b) and normalised to CS activity (f). (c–e, g, h) Superoxide dismutase and catalase are normalised to total protein (c–e) and normalised to CS activity (g, h), respectively. Western blots are representative images showing three samples in LV and LA. LV (*n* = 11), LA (*n* = 11). Data are expressed as means ± SD.

The expression levels of mitochondrial OXPHOS complexes I–V, superoxide dismutase 2 (SOD2) and catalase were significantly higher in the LV group when normalised to total protein stain (*P* < 0.01 for all, Figure 4b–d). However, these protein levels in the LV group appeared lower when normalised to CS activity (*P* < 0.01 for all, Figure 4f–h). These results suggest a higher mitochondrial abundance in the LV tissues compared to the LA; however, the LV tissue exhibits a lower expression of OXPHOS complex and antioxidant enzyme proteins per mitochondrial unit.

## 4 DISCUSSION

Given the difficulty of tissue accessibility during routine cardiac surgery, the atrial appendage offers greater practicality and a lower‐risk source of viable human tissue for metabolic studies (From et al., 2011). LA mitochondrial characteristics are often overlooked in human studies and extrapolated from atrial mitochondrial findings or vice versa in animal studies from LV findings. Human studies mainly utilise heart tissues from patients with complex disease backgrounds and medications; these factors may directly influence the data interpretations of mitochondrial function from atrial tissue to translate to LV tissues. Understanding the fundamental differences in mitochondrial function between atrial and LV chambers under healthy states is a critical foundation for meaningful comparisons to disease states, particularly when access to healthy human LV tissue is limited.

To our knowledge, our study is the first to comprehensively assess mitochondrial O_2_ flux, ATP production and hydrolysis rates, ROS production rate, OXPHOS complexes and antioxidant enzyme expression levels between the LV and LA tissues from healthy rat hearts. Per tissue mass, LV tissues showed significantly higher mitochondrial content, O_2_ flux, ATP production, ROS production, mitochondrial OXPHOS complexes and antioxidant enzyme proteins. When normalised to mitochondrial content by CS activity, the LA demonstrated higher respiratory capacity and ATP production in the OXPHOS state and consistently higher mitochondrial complex proteins and antioxidant enzyme levels. These findings suggest distinct regional adaptations: the LV supports higher energetic demands through mitochondrial quantity, while the LA compensates by enhancing functional efficiency and redox capacity per mitochondrion. Despite these differences; RCRs, P/O ratio and ROS production rates were comparable between the LV and LA tissues, suggesting comparable mitochondrial flexibility in transitioning between leak and OXPHOS respiratory states and mitochondrial energy efficiency.

### 4.1 Mitochondrial respiration

Our findings of higher O_2_ consumption rates in all respiratory states in the LV tissue are due to higher mitochondrial density, which aligns well with the higher energetic demands of the LV with its systemic contractile function. Previous studies in failing human hearts consistently show higher LV mitochondrial respiratory capacity per tissue mass compared to the LA (Lemieux et al., 2011). Normalising to CS activity, a well‐established biomarker of mitochondrial content, revealed that LV mitochondria had lower OXPHOS respiration and ATP production rates per mitochondrial unit. These findings differ from previous studies reporting no differences in chamber‐specific mitochondrial function (Krajčová et al., 2020; Lemieux et al., 2011), and may be potentially due to variability in patient pathology, clinical backgrounds and pharmacotherapy, which could affect mitochondrial function profiles.

The RCR, reflecting mitochondrial coupling efficiency in transition from OXPHOS to leak respiratory states, was not different between LV and LA tissues, suggesting that mitochondrial coupling efficiency is comparable despite chamber‐specific roles. Our findings are consistent with a previous study showing no difference in the RCR of the LA and LV in brain‐dead donor patients without any heart disease background (Krajčová et al., 2020). In contrast, earlier studies in patients with heart failure and hypertrophic cardiomyopathy have reported higher RCR in LV tissues compared to RA tissues (Scheiber et al., 2019) or RV tissues (Hamilton, 2013), suggesting that these differences reflect region‐specific pathological remodelling rather than intrinsic baseline variations in mitochondrial coupling function, at least in healthy states. Our results indicate that mitochondrial coupling efficiency is preserved between heart chambers under healthy conditions, ensuring flexibility of energy homeostasis throughout the heart regions under various energy demand conditions.

### 4.2 ATP synthesis and hydrolysis measurement

P/O ratios reflect mitochondrial efficiency in ATP synthesis coupled to O_2_ consumption, yet no studies have compared the P/O ratio between LA and LV mitochondria from healthy hearts. The P/O ratio has been well‐characterised in LV tissues from animal studies. Animal studies have reported a lower P/O ratio in STZ‐induced type 1 diabetic rat LV tissues (Pham et al., 2014) or an unchanged P/O ratio in *db*/*db* and *ob*/*ob* mice (Boudina et al., 2005, 2007) or Type 2 diabetic rats (Pham et al., 2025) when supported with glucose‐derived substrates, but the P/O ratio appeared lower when supplied with glucose plus fatty acid substrates (Boudina et al., 2005, 2007). A limitation of our current study is that we primarily focused on glucose‐derived substrates and did not include fatty acid‐derived substrates in our protocols, thus not directly assessing the overall impact of various substrates on the P/O ratio or mitochondrial respiration. ATP production was consistently reduced across these disease models. Spontaneously hypertensive rats showed an unchanged P/O ratio in LV tissues, with the same reduction in both ATP production and mitochondrial O_2_ flux (Power et al., 2016). In this study, we showed that LV tissues exhibited lower ATP production and lower O_2_ flux normalised to CS activity compared to LA tissue, and there were no differences in the P/O ratio. These findings suggest that LV mitochondria possess a lower respiratory rate per mitochondrion with comparable mitochondrial energy efficiency in healthy states. Collectively, changes in mitochondrial energy efficiency could be metabolic substrate‐dependent or pathologically dependent and are independent across heart chambers.

Real‐time ATP measurements to determine ATP consumption under anoxic conditions revealed no statistically significant differences between LV and LA tissues in mitochondrial ATP hydrolysis under anoxic conditions. A similar pattern of ATP flux during normoxia and anoxia has been observed in diabetic rat hearts, where diabetic mitochondria decreased ATP production rate in normoxic conditions but exhibited the same ATP hydrolysis rate during anoxia (Pham et al., 2014). Diabetic hearts are more susceptible to ischaemic insults (Paulson, 1997), which could be attributed to a lower ATP production rate in normoxia, not being sufficient to cover the energy deficits in anoxia. Our data suggest that LA mitochondria are more flexible in maintaining energetic cost to preserve mitochondrial function during anoxia, and with their higher ATP production rate per mitochondrion unit indicating the robustness of the LA mitochondria under stress conditions.

### 4.3 ROS production measurements

Our findings show higher ROS production per tissue mass in the LV tissue, consistent with its higher mitochondrial content. After normalisation with CS activity, similar ROS‐generating capacity was found between groups, suggesting comparable intrinsic electron leak. We noted that a few studies have reported a higher mitochondrial ROS production per tissue mass in RV tissues compared to LV tissues in healthy rats (Schlüter et al., 2018; Schreckenberg et al., 2015). The ratio of H_2_O_2_ production to O_2_ flux was also consistent across respiratory states, suggesting stable ROS handling between the LA and LV tissues and supporting that homeostasis of cardiac oxidative stress levels needs to be maintained across the heart chambers through adjustments in mitochondrial number according to energy demands.

### 4.4 Mitochondrial complex activities and endogenous antioxidants

Our findings showed that the mitochondrial OXPHOS complex proteins and antioxidant enzymes were significantly higher in the LV per tissue mass, consistent with its higher mitochondrial density. However, LV tissues showed lower protein content after normalisation for CS activity compared to LA tissues. Our results are in alignment with a previous study of copper‐adequate rats, where atrial tissue demonstrated higher SOD activity relative to the LV (Lear & Prohaska, 1997). Additionally, our results show that the LA mitochondria exhibited enhanced mitochondrial catalase, the key mitochondrial antioxidant enzyme at the individual mitochondrion level. These findings suggest that LA mitochondria possess a greater antioxidant capacity to counter any elevated ROS under stress conditions, potentially protecting mitochondrial function during recovery. The interplay between mitochondrial function and post‐injury recovery highlights the complex, chamber‐specific responses to metabolic stress.

### 4.5 Conclusion

Our findings demonstrate that the LV and LA tissues from healthy rat hearts have differences in mitochondrial function and adaptive mechanisms to anoxic conditions. The LV responds to higher energy demand by having a higher mitochondrial number. In contrast, the LA maintains energy homeostasis through more efficient mitochondria, which produce higher energy and exhibit greater antioxidant capability. Despite structural and biochemical changes, mitochondrial coupling flexibility and mitochondrial energy efficiency (P/O ratio) remain comparable between chambers. Our findings provide a foundational mechanistic understanding of future research investigating chamber‐specific differences in mitochondrial function across various cardiac disease states.

## AUTHOR CONTRIBUTIONS

All authors designed the study. Tingting Fang was responsible for the statistical analysis of data, preparing figures and drafting the manuscript. Alex Chan performed the experiments and was responsible for the data acquisition. Janice Chew‐Harris contributed to the interpretation and statistical analysis of data. Toan Pham performed the experiments and was responsible for the supervision, funding acquisition, statistical analysis of data, preparing figures and drafting the manuscript. All authors have read and approved the final version of this manuscript and agree to be accountable for all aspects of the work in ensuring that questions related to the accuracy or integrity of any part of the work are appropriately investigated and resolved. All persons designated as authors qualify for authorship, and all those who qualify for authorship are listed.

## CONFLICT OF INTEREST

None declared.

## Data Availability

The data that support the findings of this study will be made available from the corresponding author upon request.
